# How gender dysphoria and incongruence became medical diagnoses – a historical review

**DOI:** 10.1080/19585969.2022.2042166

**Published:** 2022-06-01

**Authors:** Marc-Antoine Crocq

**Affiliations:** Department of Child and Adolescent Psychiatry, Centre Hospitalier, Mulhouse, France

**Keywords:** Gender dysphoria, gender incongruence, transgender

## Abstract

This article is a historical review of the medical and psychiatric diagnoses associated with transgender people across epochs. Ancient Greek and Roman writings already mention gender change. Before a diagnosis even existed, historical documents described the lives of numerous people whom we would consider transgender today. The development of medical classifications took off in the nineteenth century, driven by the blooming of natural sciences. In the nineteenth century, most authors conflated questions of sexual orientation and gender. For example, the psychiatrist Krafft-Ebing reported cases of transgender people but understood them as paranoia, or as the extreme degree of severity in a dimension of sexual inversion. In the early 1900s, doctors such as Magnus Hirschfeld first distinguished homosexual and transgender behaviour. The usual term for transgender people was transvestite, before Harry Benjamin generalised the term transsexual in the mid-20th century. The term transgender became common in the 1970s. This article details the evolution of diagnoses for transgender people from DSM-III and ICD-10 to DSM-5 and ICD-11.

Gender Dysphoria (GD) became a psychiatric diagnosis in the fifth edition of DSM (2013), and Gender Incongruence (GI) appeared in ICD-11, the WHO classification that was approved in 2019 and should be effective in 2022. GI was not included in the section on mental health but instead in a section on sexual health. The introduction of GD and GI in today’s medical nomenclatures was hailed as progress because it intended to facilitate the provision of hormonal therapy and surgical reassignment in the context of sexual transition. However, some critics argue that gender identity is a free choice that medical authorities should not sanction. In addition, a diagnostic category is a simplistic way of describing all the nuances of gender fluidity that have asserted themselves in recent decades.

This historical review shows that the usages and connotations of terms associated with sex and gender have evolved across places and historical periods. Therefore, we should be wary of making anachronisms by understanding historical terms with our current references.

## Gender metamorphoses in Greco-Roman culture

The medical classifications that appeared in the Age of Enlightenment regarded themselves as heirs to the Greco-Roman models of antiquity. Non-binary gender and gender transformations were common themes in Greco-Roman mythology. In the Metamorphoses (the Greek μεταμορφώσεις means ‘Transformations’), the Roman poet Ovid (ca. 8 A.D.) relates how Tiresias was transformed into a woman, punished for disturbing two giant snakes mating. He lived as a woman for seven years before being turned into a man again. Once Jupiter, ‘expansive with wine and exchanging pleasantries with Juno,’ told her that women derive more sexual pleasure [voluptas] than men (Book III, 320). Juno denied it, and they agreed to ask Tiresias for his opinion since he had known sex from both perspectives. As the arbiter of the dispute, Tiresias. did confirm Jupiter’s words. Ovid further narrates (Book XII, 190) how Caenis (Καινις), a renowned beauty, was raped by Poseidon, the god of the sea. In response to Poseidon’s offer to realise a wish, she asked to be transformed into a man to prevent the repetition of such an assault. Thus, Caenis became as a man, Caeneus (Καινεύς), a famous warrior who received the additional faculty of having a skin that any weapon could not wound.

Transgender themes are not limited to mythology but are also associated with Roman emperors. According to Varner (2008), Roman imperial portraits could be quite gender fluid and even transgender, consciously hybridising elements of traditional male and female categorizations. In imperial portraits, the mixture of human and divine, male and female, intentionally blurred traditional taxonomic categories to assert the transcendence of imperial authority over prescribed gender roles. For example, museums contain busts, coins, gems showing the identification of emperor Domitian with the female goddess Minerva. These artefacts show Minerva’s recognisable female body or hairstyle mixed with Domitian’s facial features.

The Roman emperor Elagabalus (ca. 204–11 March 222), also called Heliogabalus, might be the first historical record of a famous person asking to be addressed femininely and requesting surgical sexual reassignment. According to Cassius Dio (c. 155–c. 235 AD) (Dio Cassius: Roman History 1955), Elagabalus once said: ‘call me not Lord (κύριος), for I am a Lady (κυρία).’ Also, he asked physicians to contrive a woman’s vagina in his body through an incision, promising them large sums for doing so. Elagabalus was just an adolescent when he became an emperor and hardly an adult when assassinated. Cassius Dio, a statesman and historian who wrote in Greek was a contemporary of Elagabalus. However, he was not a direct witness since he was in Pergamum and Smyrna during most of Elagabalus’ reign, which he generally depicted as a period of decline (Scott 2018).

## Transgender behaviour existed before diagnostic classifications

Historical sources from the early modern period in Europe indicate that cases of persons living permanently with the clothes and roles of the other sex were not exceptional. History has highlighted the most adventurous biographies. Catalina de Erauso (Repubblica) ran away from her convent in Spain in the early seventeenth century as a young woman. She adopted a male identity and embarked to America, where she was active in business or the military in almost all the provinces of the Spanish Empire. She is said to have posed as a castrato – a legitimate social status at the time – to pursue love relationships with feminine partners. She achieved such fame that she gained audiences with the king of Spain, Philip IV, and the Pope, Urbano VIII, who granted her permission to wear male clothes. She died in Mexico under the name Antonio de Erauso. Another exceptional biography is the Chevalier d’Éon, who served the French King Louis XV as an envoy and spy in Russia and as an officer on the Seven Years’ War battlefields. He spent the last part of his life in England as *Mademoiselle* or *Chevalière* d’Éon, dressing as a woman. His birth sex remained a mystery and was the subject of speculation until his death. Only then did a surgeon attest in a post-mortem certificate that he had well-formed male genital organs.

## Early medical characterisations. The conflation of homosexual and transgender

The current psychiatric classifications were initially developed by German-speaking authors in the nineteenth century. At that time, homosexuality, transvestism, or transgender behaviours were conceptualised as various degrees of a single dimension of ‘gender/sexual inversion.’ This conflation of homosexuality and gender contrasts with modern notions that homosexuality is independent of gender identity and related exclusively to sexual object choice.

The essential point in Karl Heinrich Ulrichs’ theory of homosexuality (1864) was the conviction that the male homosexual possesses a female soul enclosed in a male body (‘*anima muliebris in corpore virili inclusa*’). Ulrichs believed that both possibilities of sexual development remained possible in the embryo’s early stages when the sexual organs were not yet differentiated. He saw confirmation of this idea in the existence of hermaphrodites. He postulated that there must be a ‘germ’ [‘*Keim*’] that determined whether the sexual organs would develop male or female. To explain the discrepancy between the sexual organs and the sexual orientation, he postulated the existence of another ‘germ’ that determined the direction of the sex drive. We will see that this model prefigures modern biological conceptions. The term homosexuality was coined in 1869 by the Austro-Hungarian writer Karl Maria Kertbeny.

Krafft-Ebing (1840–1902), influenced by the theory of degeneracy, postulated a gradient of severity from effeminacy and homosexuality to complete *transmutatio sexus* [sex transmutation]. In the 7th edition of his textbook of sexology, *Psychopathia sexualis*, he gives a clinical description that would satisfy the DSM-5 criteria of GD. The person referred to as Case 99 (von Krafft-Ebing 1894; or Case 129 in the textbook’s 12^t^h edition), a physician born as a male in Hungary in 1844, writes autobiographically:

at the age of twelve or thirteen, I had a definite feeling of preferring to be a young lady. A young lady’s form was more pleasing to me; her quiet manner, her deportment, but particularly her attire, attracted me. But I was careful not to allow this to be noticed. I am sure that I should not have shrunk from the castration-knife, could I have thus attained my desire. If asked why I preferred female attire, I could have said nothing more than that it attracted me powerfully… I remember, when fifteen, to have first expressed to a friend the wish to be a girl. In answer to his question, I could not give the reason why … when in high school I finally had once coitus; *hoc modo sensi, me libentius sub puella concubuisse et penem meum cum cunno mutatum maluisse* (litteraly: I would have preferred to lie under the girl and to have her sexual organs exchanged for my penis) … But, even on my marriage-night, I felt that I was only a woman in man’s form.

Krafft-Ebing diagnosed this case as ‘*Metamorphosis sexualis paranoiaca*’ indicating that he understood it as delusional.

## Distinguishing transvestite, transsexual, and transgender

Before World War I in Berlin, Magnus Hirschfeld first distinguished between the questions of sexual orientation and those of gender identity, putting an end to the confusion between the fields of homosexuality and gender identity. However, Hirschfeld still used the word transvestite to refer to what we call transgender today. After the World Wars in the United States, Harry Benjamin clearly defined the difference between transvestite and transsexual. After the 1970s, the term transsexual gave way to transgender (Yarbrough 2018).

Magnus Hirschfeld (1868–1935) was a physician who practiced in the Charlottenburg district of Berlin. He founded two organisations committed to the support of homosexuals and sexual minorities, first the *Wissenschaft-humanitäres Komitee* (WhK) in 1897, and then the *Institut für Sexualwissenschaft* (1919) whose staff included transgender people (Bauer et al. 2017). Hirschfeld published an annual journal entitled ‘*Jahrbuch für sexuelle Zwischenstufen unter besonderer Berücksichtigung der Homosexualität*’ (Yearbook for sexual intermediaries with special reference to homosexuality), which aimed to spread scientific research to carry out advocacy on behalf of sexual minorities (Dobler, 2004). One of his objectives was the revision of paragraph 175 of the German criminal code, which criminalised homosexual acts between males. As recounted by Susan Stryker (2017), Hirschfeld had a central position in the history of the transgender movement. He collaborated with Eugen Steinach (1861–1944), the Austrian endocrinologist who worked on the anatomical and behavioural effects of sexual hormones (Steinach 1912). One of his young colleagues was Harry Benjamin, who settled in the United States. Two transgender women seen by Hirschfeld in the late 1920s et early 1930s belong to the first documented cases of sex reassignment surgeries: Dora [Dörchen] Richter (1931), and the Danish painter Lili Elbe whose life was fictionalised in the film *The Danish Girl*. As a Jew and homosexual, Magnus Hirschfeld had to leave Germany and died in exile in France.

According to Drescher (2014), Hirschfeld is credited with being first to distinguish the desires of homosexuality (to have partners of the same sex) from those of transsexualism (to live as the other sex), thus putting an end to the conflation of gender identity and sexual orientation. He used the term ‘transvestites’; he also coined the term ‘transsexual’ in 1923 but this word would only catch three decades later with H. Benjamin. Hirschfeld’s famous book-length publication on transvestites appeared in 1910 in the *Jahrbuch* (Hirschfeld 1910). A sequel publication illustrated with numerous drawings and photos of transvestites, historical or contemporary, was released in 1912 (Hirschfeld and Tilke 1912). According to Marhoefer (2015), Hirschfeld’s contemporaries disagreed over whether ‘transvestites’ denoted people who only wished to dress in the clothing of the other sex, or people whose actual sex was not their birth sex and who transitioned to their actual sex, or both of these groups. Three decades later, the former would be called ‘transvestites’ and the latter ‘transsexuals’. Mak (1998) remarks that Hirschfeld included only one woman in his transvestite case histories. In an article based on the cases of four women whom Hirschfeld knew while writing his book on transvestites, Maak hypothesises that Hirschfeld continued to regard the ‘masculinity’ of feminists as a hallmark of inverted sexual identity, an ancient model which had flourished in the nineteenth century.

Havelock Ellis (1859–1939), a British physician, also studied transgender phenomena as a question distinct from homosexuality. He disagreed with Hirschfeld’s term ‘transvestitism’ and proposed in 1913 the term *sexo-aesthetic inversion* instead (Ekins and King 2006). In the 1920s, he coined the word *eonism*, which he derived from the name of the historical figure Chevalier d’Éon.

Harry Benjamin visited the United States in 1913. As fate would have it, the First World War broke out shortly after that, and the Royal Navy blockade cut him off from the way back to Germany. He lived in the United States till the age of 101-½ (Green 2009). Guided by his interest in hormonal research, he became a disciple of Eugen Steinach, whom he visited in Vienna every summer through the twenties and early thirties. On these occasions, he also took frequent trips to Berlin, where he would meet Magnus Hirschfeld. He also knew Alfred C. Kinsey, who acquainted him in 1948 with a young patient, Barry. Born with male sex, Barry started dressing in girls’ clothes by age 3. He denied ever having an erection or even masturbating. Medical people were advising genital surgery, but conversion intervention was prevented by the Attorney General of Wisconsin and state laws interpreting such surgery as ‘mayhem’. Through Benjamin’s encouragement, Barry, now known as Sally, made three trips to Europe between 1953 and 1958 to complete the genital operations, which culminated in the construction of a vagina lined with skin from the thigh (Schaefer and Wheeler 1995).

Benjamin’s most famous patient, George Jorgensen, went to Denmark as a natal man and returned to the US in 1952 as trans woman Christine Jorgensen. When she died at 62 in 1989, a New York Times obituary retraced her biography. Growing up, she was frustrated by feelings that she was a woman trapped in a man’s body. She served as a clerk in the army and was honourably discharged. Her sexual conversion began with hormone injections in 1950 when she was 24 years old and was completed in 1952 with surgery in Copenhagen under the care of Christian Hamburger, a Danish specialist whose first name she adopted to form her own. Hamburger and his colleagues published a report in the Journal of the American Medical Association (Hamburger et al. 1953).

Such cases led to Benjamin’s interest in what he later described as transsexualism, realising that there was a different condition to that of transvestism, which was then the current diagnosis for adults who had such needs. Benjamin thought that

transvestitism is the desire of a certain group of men to dress as women, or of women to dress as men … It can be overwhelming, even to the point of wanting to belong to the other sex and correct nature's anatomical ‘error’ … For such cases the term transsexualism seems appropriate. True transsexuals have the feeling that they belong to the other sex; they want to be seen and function as members of the opposite sex and not only appear as such. Their sexual organs, primary (testicles) and secondary (penis and others), are disgusting deformities to be changed by the surgeon's scalpel. It is only because of the recent and great advances in endocrinology and surgical techniques that the picture has changed. (Benjamin 1953)

Another historical pioneer is Michael Dillon (1915–1962). Born as a girl in London, he started self-administering testosterone tablets in 1939. He underwent his first operation for phalloplasty in 1945. He graduated from medical school at Trinity College in Dublin in 1951. He finished his life in India as a Buddhist monk under the name Lobzang Jivaka. His autobiography, *Out of the Ordinary*, was published posthumously in 2017. He has been called the first female to male ‘transsexual,’ although that term would become common only after his lifetime.

Today, expressions of gender variance or gender nonconformity are frequently subsumed by the popular term transgender, although this term does not appear in the DSM or ICD. ‘Transgender’ is a relatively recent word. According to the Oxford English Dictionary, ‘transgender’ was first used in the early 1970s to designate a person whose sense of personal identity and gender does not correspond to that person’s sex at birth or does not otherwise conform to conventional notions of sex and gender. It is also an umbrella term which includes any or all non-conventional gender identities.

## Psychology, society, or biology?

A diagnostic category needs an aetiological hypothesis to enhance its validity. Authors have been searching in three main fields for influences that might make a person transgender: psychology, sociology, and biology. A proponent of the role of sociology and education was John Money, a New Zealand psychologist. He published theories in the 1950s that one’s sense of being male or female was acquired and determined by external, environmental factors. Based on cases of gender assignment in intersex children born with ambiguous genitalia, Money believed parental attitudes had a substantial effect on whether a child accepted the gender category that had been surgically and medically assigned.

Benjamin is quoted as having little regard for his era’s psychoanalysts and believing that transgender women had a brain that was probably ‘feminized’ *in utero*. This belief seemed to be confirmed by a milestone paper published in *Nature* in 1995 (Zhou et al. 1995) that studied the volume of the central subdivision of the bed nucleus of the stria terminalis (BSTc). The BSTc, a brain area essential for sexual behaviour, is more prominent in men than women. A female-sized BSTc was found in male-to-female transsexuals. The size of the BSTc was not influenced by sex hormones in adulthood and was independent of sexual orientation. According to the authors, this study was the first to show a female brain structure in genetically male transsexuals and supported the hypothesis that gender identity develops due to an interaction between the developing brain and sex hormones. Swaab (2014), the senior author, further elaborated that the differentiation of our sex organs occurs in the first months of pregnancy. In contrast, the sexual differentiation of the brain occurs in the second half of pregnancy. One might hypothesise that these two successive differentiations may occur in different directions in some transgender persons. This hypothesis is reminiscent of Ulrichs’ theory mentioned earlier (vide supra).

Recent articles suggest that the sexual differentiation of the human brain is more complex than initially thought. MRI analyses of approximately 1500 human brains showed extensive overlap between the distributions of females and males for all parameters assessed. Moreover, brains with features that are coherently at one end of the ‘maleness-femaleness’ continuum are rare. Instead, most brains comprise unique ‘mosaics’ of features, some more common in females compared with males, some more common in males compared with females, and some common in both females and males (Joel et al. 2015). However, recent studies comparing transgender and cisgender persons show differences in the cerebral networks involved in own body perception in the context of self (Manzouri and Savic 2019; Nota et al. 2017), and in the patterns of brain connectivity that might affect one’s sense of body congruence (Moody et al. 2021). If proven, a biological aetiology would destigmatize transgender behaviour.

## DSM and ICD

Table 1 shows the diagnostic categories associated with transgender persons in the successive editions of DSM and ICD. Drescher has finely analysed the evolution of these diagnoses in DSM (Drescher 2020). The first two editions of the DSM placed a significant emphasis on psychoanalytic theories of normal and pathological mental functioning; the gender identity diagnoses or anything equivalent did not appear in either one (APA, 1952, 1968).

**Table 1.** Gender as a diagnostic category in DSM and ICD.

Diagnostic classification and year	Diagnostic terms	Comments
DSM-II 1968	No diagnostic category for gender incongruence	‘Transvestitism’ is included in the sexual deviations
DSM-III 1980	Transsexualism, with subclassification as asexual, homosexual, or heterosexual, and with reference to the sex assigned at birth. Gender Identity Disorder of Childhood (GIDC)	Transvestism is a differential diagnosis GIDs are in the section on Psychosexual Disorders, together with Paraphilias, Psychosexual Dysfunctions, and Ego-dystonic Homosexuality
DSM-III-R 1987	Gender Identity Disorder of Childhood Transsexualism Gender Identity Disorder of Adolescence or Adulthood, Nontranssexual Type (GIDAANT)	GIDAANT: for cross-gender identified individuals who did not pursue reassignment.
ICD-10 1990	Transsexualism, duration ≥ 2 years Dual-role transvestism Gender identity disorder of childhood (no specific duration requirement)	Fetishistic transvestism is a diagnostic category in the ‘Disorders of sexual preference.’
DSM-IV(-TR) 1994 (2000)	Gender Identity Disorder. Children are coded differently than Adolescents or Adults. Specify if sexually attracted to males, females, both, or neither	GIDs are included in the section on Sexual and Gender Identity Disorders, which also contains Sexual Dysfunctions and Paraphilias.
DSM-5 2013	Gender Dysphoria in Adolescents and Adults Gender Dysphoria in Children. A minimal duration of 6 months is required for both diagnoses.	GD is placed in a distinct section. The terms Gender and Sex are defined and used differently. Contrary to DSM-IV, sexual orientation is not specified.
ICD-11 2019-2022	Gender Incongruence of adolescence or adulthood. several months. Gender Incongruence of Childhood. In children, the incongruence must have persisted for about 2 years.	Included in the chapter on ‘Conditions related to sexual health’ ICD-11’s primary focus is experience of incongruence between experienced gender and assigned sex; In ICD-11, distress and functional impairment are described as common associated features, particularly in disapproving social environments, but are not required; in contrast, DSM-5 requires clinically significant distress or impairment for diagnosis. Body Integrity Dysphoria is a differential diagnosis

### DSM-III to DSM-IV-TR

Zucker and Spitzer (2005) summarised the mutations of the gender diagnoses from DSM-III through DSM-IV-TR. In the DSM-III (1980), there appeared for the first time two psychiatric diagnoses in children, adolescents, and adults: gender identity disorder of childhood (GIDC) and transsexualism, the latter concerning adolescents and adults. The decision to place transsexualism in the DSM was supported by the research and clinical contributions of John Money, Harry Benjamin, Robert Stoller, and Richard Green. A third diagnosis appeared in the DSM-III-R (APA, 1987): gender identity disorder of adolescence and adulthood, nontranssexual type (GIDAANT). However, GIDAANT was not kept in DSM-IV (APA, 1994, 2000a), and GIDC and transsexualism were collapsed into one overarching diagnosis, gender identity disorder (GID), with different criteria sets for children versus adolescents and adults.

### ICD-10

The ICD-10, endorsed by the Forty-third World Health Assembly in 1990, followed the DSM-III’s lead and included the diagnoses of transsexualism and gender identity disorder of childhood. ICD-10 also created the diagnosis of dual-role transvestism (‘*Transvestisme bivalent*’ in French). In ICD-10, Transsexualism designated a desire to live and be accepted as a member of the opposite sex, usually accompanied by a sense of discomfort with, or inappropriateness of, one’s anatomic sex and a wish to have hormonal and surgical treatment to make one’s body as congruent as possible with the preferred sex. To qualify for this diagnosis, the transsexual identity had to persist for at least two years. Dual-role transvestism designated the wearing of clothes of the opposite sex for part of the individual’s existence to enjoy the temporary experience of membership of the opposite sex, but without any desire for a more permanent sex change or associated surgical reassignment. No sexual excitement accompanied the cross-dressing, which distinguished the disorder from fetishistic transvestism.

### DSM-5

In DSM-5, being transgender is not considered per se a psychiatric disorder; it is only the resultant dysphoria that justifies a diagnosis. While dysphoria was chosen for the diagnostic title, the word incongruence is used for the definition in criterion A. In DSM-IV-TR, criterion A requested ‘cross-gender identification.’ In DSM-5, this criterion was modified in order to avoid a male-female dichotomy, and the expressed gender can be female, male, in-between, or otherwise. The primary reason for creating separate diagnostic categories for GD in children on the one hand, and adolescents and adults on the other, is a difference in course. The longitudinal observation of children with GD shows that the persistence of GD into adolescence and adulthood is variable, ranging from 2% to 50% (Zucker et al. 2013). On the other hand, in adolescents and adults, there is considerable evidence of diagnostic stability.

In DSM-5, the term transvestic has nothing to do with gender. Transvestic Disorder is part of the section on paraphilias. The diagnosis of transvestic disorder applies to individuals whose cross-dressing or thoughts of cross-dressing are always or often accompanied by sexual excitement. The specification ‘with fetishism’ indicates that fabrics, materials, or garments sexually arouse the person. The specification ‘with autogynephilia’ indicates that thoughts or images of self as female provoke sexual arousal. According to Blanchard (2010), the presence of fetishism decreases the likelihood of GD in men with transvestic disorder, whereas autogynephilia increases the likelihood of GD in men with transvestic disorder. Individuals with transvestic disorder do not report an incongruence between their experienced gender and assigned gender or desire to be of the other gender. Individuals with a presentation that meets full criteria for transvestic disorder and GD should have both diagnoses.

### ICD-11

The diagnosis of gender incongruence (GI) was included in the ICD-11 to preserve access to health services, but it was moved from the ICD-11 chapter on Mental and Behavioural Disorders to the chapter on Sexual Health. Following DSM-5, The ICD-11 abandoned ICD-10 terms such as ‘opposite sex’ and ‘anatomic sex,’ using more contemporary and less binary terms such as ‘experienced gender’ and ‘assigned sex.’ Unlike ICD-10, but like DSM-5, the proposed ICD-11 diagnostic guidelines do not implicitly presume that all individuals seek or desire complete transition to the ‘opposite’ gender. In ICD-11 (Reed et al. 2016), GI is characterised by a marked and persistent incongruence between an individual’s experienced gender and the assigned sex. Gender variant behaviour and preferences alone are not a basis for assigning GI diagnoses in this group, whether in adolescents, adults, or children.

The ICD-10 categories fetishistic transvestism and dual-role transvestism disappeared in ICD-11. These conditions involve consensual or solitary sexual activity, cause no inherent harm to self or others, and are not necessarily distressing to the individual or associated with functional impairment. Therefore, these arousal patterns were not considered per se as mental disorders but more accurately as variants in sexual arousal (Krueger et al. 2017).

DSM-5 emphasises distress and dysfunction related to gender identity through the category’s name and criteria. Distress and dysfunction are also the central rationales for classifying gender dysphoria as a mental disorder. In CIM-11, the diagnostic guidelines indicate that GI may be associated with clinically significant distress or impairment in social, occupational, or other important areas of functioning, particularly in disapproving social environments and where protective laws and policies are absent, but that neither distress nor functional impairment is a diagnostic requirement.

## From diagnosis to depathologization

The theme of gender change is frequent in ancient Greco-Roman sources. The reliable historical documents available in the premodern era produced numerous and continuous testimonies of people who led lives that would seem transgender today. The interest in medical diagnosis started in the mid-nineteenth century, thanks to expanding natural sciences. In the twentieth-century culture, giving a medical diagnosis is double-edged, allocating both help and pathological labelling to the person. The concern for depathologization guides the most recent classifications. The aetiology of transgender behaviours is unknown, but research suggests that they have a biological basis in the brain. If this is the case, transgender expression is not a matter of choice influenced by social fads. Nor is it a matter that must be subject to religious or political rules.
